# Area-Controlled Soft Contact Probe: Non-Destructive Robust Electrical Contact with 2D and Fragile Materials

**DOI:** 10.3390/ma17051194

**Published:** 2024-03-04

**Authors:** Michiko Yoshitake, Kaori Omata, Hideyuki Kanematsu

**Affiliations:** 1 National Institute for Materials Science (NIMS), Tsukuba 305-0047, Japan; 2 National Institute of Technology (KOSEN), Suzuka College, Suzuka 510-0294, Japan; kaori.o@yamanashi.ac.jp (K.O.); kanemats@suzuka.kosen-ac.jp (H.K.); 3 Graduate Faculty of Interdisciplinary Research, University of Yamanashi, Yamanashi 400-0016, Japan; 4 Institute of Innovation for Future Society, Nagoya University, Nagoya 464-8601, Japan; 5 Division of Materials & Manufacturing Science, Graduate School of Engineering, Osaka University, Osaka 565-0871, Japan

**Keywords:** electrical contact, soft probe, 2D (two-dimensional) materials, fragile materials, contact area control

## Abstract

We developed a soft contact probe capable of making electrical contact with a specimen without causing damage. This probe is now commercially available. However, the contact area with the probe changes according to the pressure applied during electric contact, potentially affecting electric measurements when current density or electric field strength is critical. To address this, we developed methods to control the area of electric contact. This article reports on these methods, as well as variations in probe size, pressure for electric contact, probe materials, and attachment to commercial probers.

## 1. Introduction

**Figure 1:**
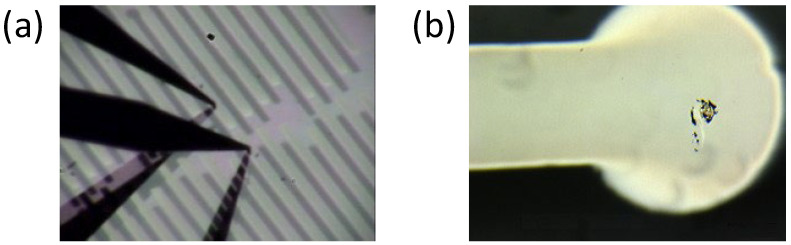
(**a**) Electrical contact with usual needle-type probers and (**b**) damage on an electrode by the contact.

Electrical measurements of emerging materials, such as 2D materials [1,2,3,4,5,6] and fragile materials like MOFs [7,8,9,10,11], are crucial for developing devices using these materials. A commercially available prober that uses metal needles as a probe, such as in Figure 1a, scratches specimens and damages the surface, as seen in Figure 1b. Therefore, metal evaporation [1,12,13] or conductive AFM (atomic force microscopy) [14,15,16,17] has usually been employed to make electrical contact with specimens. However, these methods have drawbacks: metal evaporation contaminates specimens, preventing further measurements, and AFM requires precise feedback. Additionally, some materials suffer thermal damage during metal evaporation [18,19,20,21], so cooling during evaporation was needed [22]. The penetration of evaporated metal through organic molecules is also problematic [23,24,25,26].

**Figure 2:**
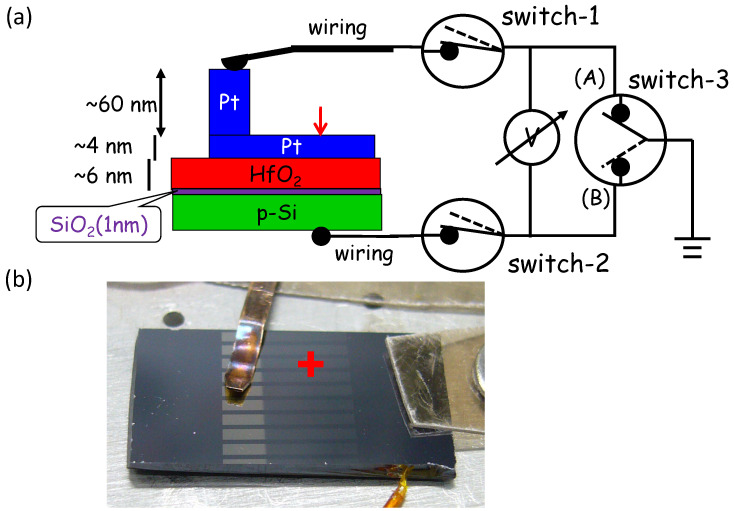
(**a**) The schematical shows structure of the MOS specimen, the location of analysis point (red arrow and cross) with an electric circuit, for biased (variable voltage) XPS measurement by changing ground potential with switch-3 between (A) and (B) [29], (**b**) photo of the specimen.

We developed an alternative: a soft contact probe that enables robust electrical contact to nm-thick films, 2D materials, or fragile specimens without contaminating them with electrode metals [27,28]. Our original motivation to develop a soft probe was operand measurement of a high-k MOS device by XPS, where we observed peak shifts under a bias voltage applied across multiple interfaces, as shown in the left side of Figure 2a. To observe peaks from the bottom Si, the top Pt gate electrode should be ultra-thin (~4 nm), enabling photoelectrons to escape to a vacuum. To avoid damaging the ultra-thin Pt film upon electrical contact, we needed to prepare a specially designed specimen, as shown in Figure 2 [29]. Here, the thickness of Pt film at contact is thick (~60 + 4 nm), while the measurement point (indicated with a red arrow in the figure) is 4 nm. However, preparing such specially designed specimens on each occasion consumes too much time and effort. To solve this problem, a soft probe that can directly make electrical contact with an ultra-thin film without damaging the specimen was developed and demonstrated the effectiveness [27] by operand measurement of the same high-k MOS device used in ref. [29].

This probe type, available in various lengths to control the pushing pressure, is now commercially available [30]. It has been successfully used in diverse applications, such as graphene measurements under magnetic fields at low temperatures in vacuum chambers [31]. These probes are advantageous as they vibrate, have thermal expansion, and are drift tolerant due to their design, which compensates for small displacements between the specimen and the co-axial connector [31].

Despite these advances, additional aspects of the soft probe, such as variations in contact ball size, wire thickness, material types, and connection mechanisms to commercial probers, have not been fully published. This article discusses these aspects and presents experimental results on a newly developed method to control contact areas.

## 2. Concept of Non-Destructive Robust Electrical Contact

**Figure 3:**
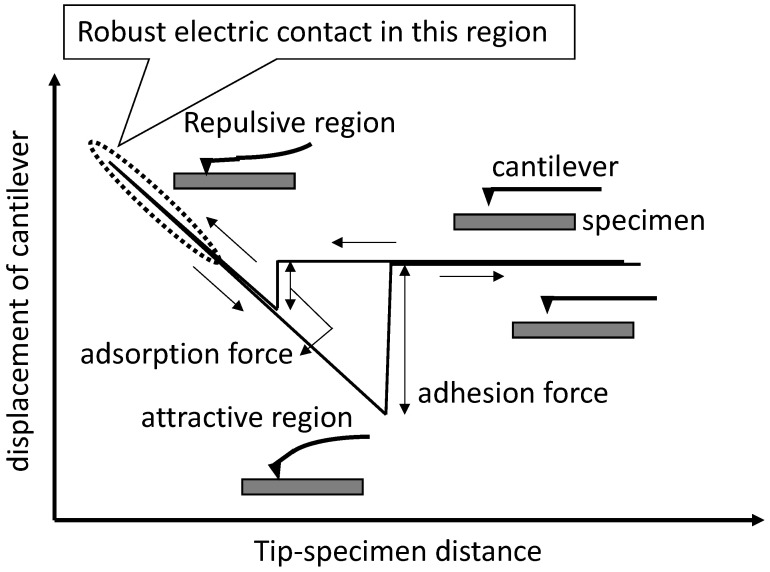
AFM force curve and relative position between a cantilever and a specimen during the force curve measurement. Electrical contact is achieved at the repulsive region in the figure.

The principle of non-destructive electrical contact relies on the physics underlying the non-destructive measurement mode in atomic force microscopy (AFM) [14,32,33]. Figure 3 depicts a typical force curve in AFM. In the repulsive region, electrons from both the tip and the sample overlap, and I-V characteristics are measured. A force curve in the repulsive region usually shows a repeatable trace as the cantilever approaches and retracts. This indicates elastic deformation in the repulsive region and complete recovery of both the sample and the tip, qualifying it as a non-destructive method. Non-destructive electrical contact is thus achieved when two materials are in contact within the repulsive region but still within the elastic deformation range.

A typical force applied in the repulsive region was calculated using a force curve on copper (Cu), measured with a cantilever having a force constant of 3 [N/m]. The calculated pressure is approximately 5 × 10^7^ [Pa], derived from {3 [N/m] × 5 [nm]}/{π × 10 [nm^2^]}. This pressure corresponds to an applied force of 1.5 [N] over a contact area of a circle with a radius of 100 [μm]. Using a commercially available spring plate with a thickness of 0.5 [mm], the displacement with a force of 1.5 [N] is approximately 1 mm, which is easily observable by the naked eye. Consequently, one can control the pressure by simple visual monitoring or using a conventional camera without the need for precise sensors.

## 3. Fabrication of a Probe for Non-Destructive Electrical Contact

### 3.1. Probe Fabrication

#### 3.1.1. Basic Probe

**Figure 4:**
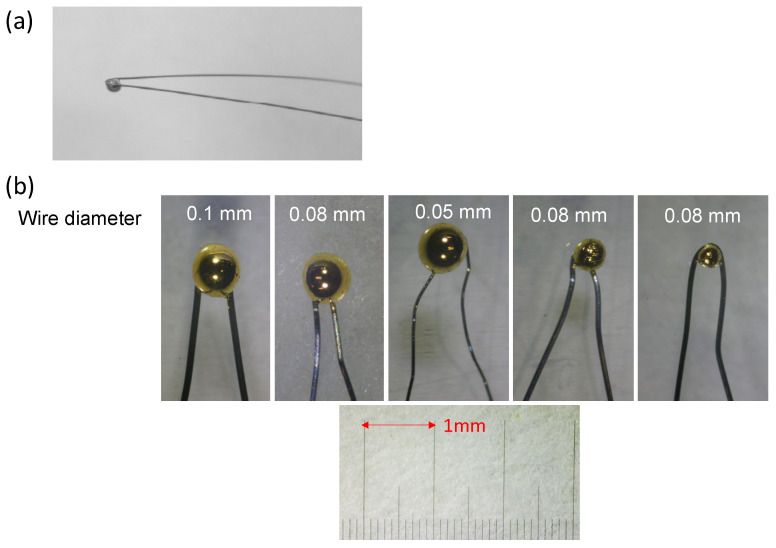
(**a**) An example of a developed contact probe, (**b**) photograph of variation examples of developed contact probes, with the sequence of contact ball size (left three are wire diameter variations with the same ball size, while the right two are ball size variations with the same wire diameter).

Figure 4a displays a photograph of a probe. This probe consists of a 70 mm long tungsten wire, 0.10 mm in diameter, bent into a hairpin shape. A 10.2 mm long gold wire, 0.15 mm in diameter, was attached at the tungsten wire’s bend. The assembly was then resistively heated in a vacuum to melt the gold, forming a hemi-spherical contact ball. This method was chosen for its ability to produce a smooth surface, minimizing damage to the specimen during electrical contact. The resulting gold ball had an approximate diameter of 700 μm, with the contact area’s radius estimated to be between 100 and 150 μm, as observed through optical microscopy. The gold ball’s size can be varied by adjusting the amount of gold wire used before melting in the vacuum.

#### 3.1.2. Variations on Contact Ball Size and Wire Thickness

The size of the contact ball and the thickness of the supporting wire can be varied, as discussed in ref. [34]. Figure 4b illustrates some of these variations. The left three in the figure are wire diameter variations with the same ball size (~φ700 μm), while the right two are ball size variations with the same wire diameter. To achieve non-destructive robust electrical contact, a probe with an optimal contact area and spring constant is necessary. The contact area is selected based on factors such as specimen size, which then determines the contact ball’s size. The wire’s thickness and length are key factors in controlling the support’s spring constant, further discussed in Section 3.2.

#### 3.1.3. Material Variations

While Figure 2 predominantly features gold balls, the material for the contact ball is not restricted to gold, as mentioned in ref. [35]. We have successfully fabricated contact balls using copper and aluminum, materials with relatively lower melting points. For balls made of materials with higher melting temperatures or those that are volatile, a deposition method onto the gold ball is effective. This technique was applied to create platinum balls.

### 3.2. Attachment to a Commercial Co-Axial Connector

**Figure 5:**
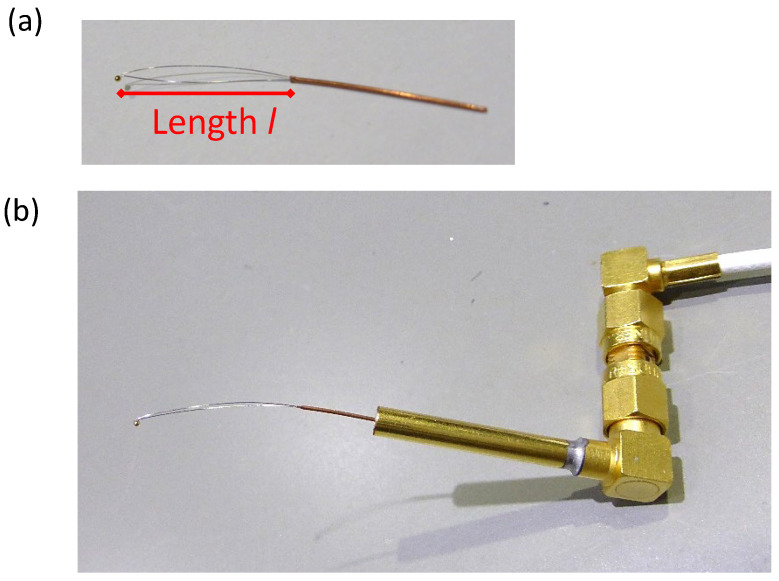
Photograph of the attachment system of a developed contact probe to a commercially available prober. Wire with a ball is inserted into a copper pipe (**a**), and the copper pipe is inserted into a co-axial connector (**b**).

To integrate the developed probes with a commercial co-axial connector for electrical measurements, we utilized a copper pipe with inner and outer diameters of 0.03 mm and 0.05 mm, respectively. Figure 5a displays a probe inserted into the copper pipe, and Figure 5b shows the probe-integrated copper pipe connected to a commercial co-axial connector. The probe’s length, as seen in Figure 5a, is a critical factor in determining the deflection under a given pressure, alongside the support wire’s thickness.

### 3.3. Amount of Deflection under a Certain Pressure on the Probe

The amount of deflection is calculated from equations in mechanical engineering textbooks [36,37,38]. For a cantilever of length *l*, the deflection is given by (F*l*^3^/(3EI), where F is a load at the cantilever’s end, *E* is Young’s modulus of the wire material (W), and I the second moment of the area. The second moment of the area for a wire with a diameter of *d* is written as (π*d*^4^)/64. In this probe, since the free edge of the probe is supported by two wires, the second moment of the area is twice the value mentioned above. Therefore, the amount of deflection of the probe is (32F*l*^3^)/(3π*Ed*^4^), with *l* being the length of the probe shown in Figure 5a. The desired reflection range is 1~5 mm, noticeable to the naked eye. By adjusting the length of the probe when inserted into the copper pipe, the same probe can be adapted for specimens of varying fragility.

### 3.4. Examples of Measurements with the Probes

**Figure 6:**
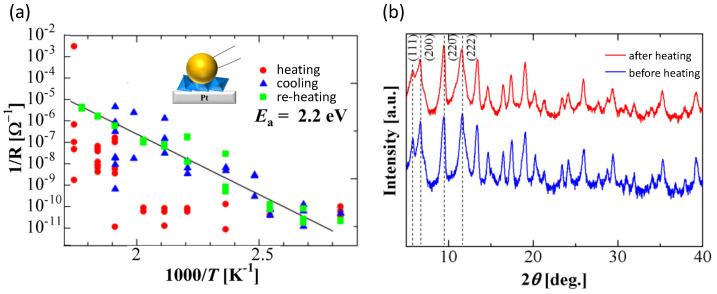
Temperature dependence of electrical conductivity (inverse of resistance, R) of MOF specimen measured with a developed soft probe (**a**). XRD pattern of the specimen of the specimen before and after heating for the measurements (**b**).

Two examples of the measurement with practical values using this type of probe are resistance measurement of a 5-layer graphene and operand XPS measurement of an MOS structure (contacting at the position indicated by the red arrow and cross in Figure 2a) bias voltage being applied via this type of probe, were demonstrated in ref. [27]. In addition, the temperature dependence of conductivity (inverse of the resistivity) of a MOF specimen (HKUST-1: Cu_3_(C_9_H_3_O_6_)_2_) was measured using this type of probe by one of the users [39,40]. Figure 6a displays the results of the measurements, where how the probe was used is illustrated inside the figure. The activation energy of the electron carrier was calculated to be 2.2 eV from the slope in the figure. At the first heating, temperature dependence shows a strange jump at ~2 (1000/T), while at cooling and re-heating, temperature dependence exhibits a similar trend with a similar slope. Since there was no change in XRD diffraction before and after the heating, as demonstrated in Figure 6b, the strange jump at the first heating seems to have an artificial effect with contact.

## 4. Contact Area Control by Lithography

### 4.1. Process Optimization of Lithography

**Figure 7:**
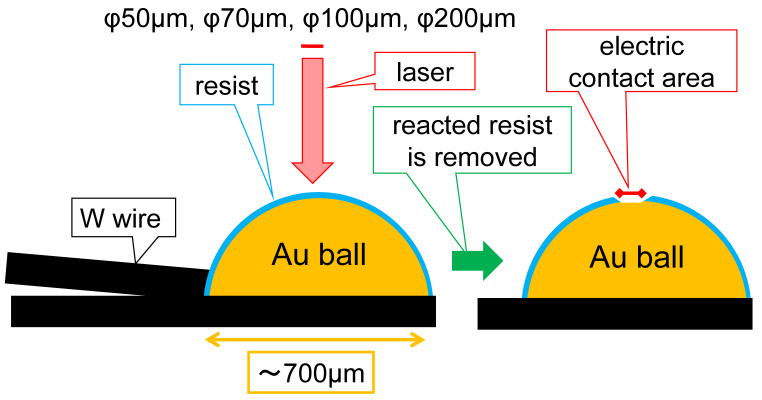
A schematic procedure of the fabrication of area-controlled probes with laser lithography.

The contact area was precisely controlled using laser lithography techniques [41,42,43,44,45,46,47,48,49]. Initially, a bare probe was dip-coated with a resist film (AZ5214E:PGMEA = 1:1), and subsequent pre-treatment, either vacuum drying or pre-baking at 110 °C for 10 min. The pre-treated probe was then exposed to laser light (NanoSystem Solutions, Inc., Katsuren Haebaru Uruma-City, Okinawa, Japan, DL-1000/NC2P, 405 nm LED, 1 W/cm^2^, 140 mJ/cm^2^) to create a round pattern [50,51]. Following this, the probe underwent development using TMAH2.38% for 90 s, a rinse in deionized water for 30 s, drying with N_2_ blow, and one of the following three post-treatments: none, post-baking at 110 °C for 10 min, or post-hard-baking at 180 °C for 20 min. Figure 7 illustrates the brief process of laser lithography. Table 1 summarizes the various pre- and post-treatment conditions used.

**Figure 8:**
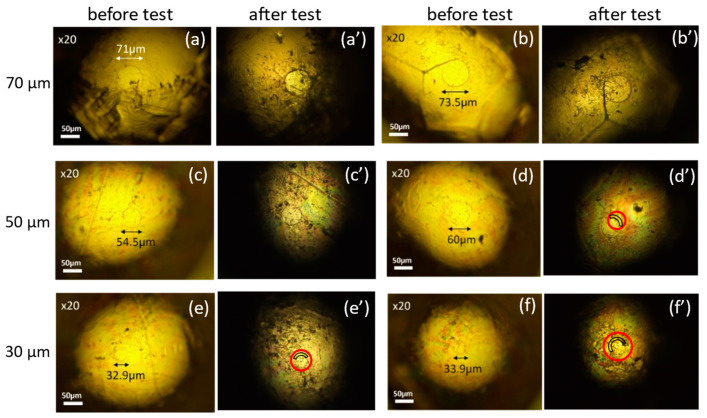
Photos of area-controlled (φ70, φ50 φ30 μm) probes with laser lithography using process #1 before (**a**–**f**) and after (**a’**–**f’**) robustness test. Inside the red circles, resist films are partially missing.

We examined the robustness of probes fabricated under various conditions with different contact area sizes: φ200 μm, φ100 μm, φ70 μm, φ50 μm, and φ30 μm. Since the results of the robustness test for probes with φ200 μm and φ100 μm are the same as those of φ70 μm, only the results of φ70 μm, φ50 μm, and φ30 μm are displayed here. The robustness was evaluated by observing the damage of the resist film after several contacts with a gold plate. Figure 8 compares photographs of probes before and after the robustness test fabricated with process #1 in Table 1 (vacuum dry and no post-treatment). For each size, two examples are shown in the figure. For φ70 μm, photos of one example before and after the test are labeled (a) and (a’), and those of the other example before and after the test are labeled (b) and (b’). A similar labeling rule applies for probes of φ50 μm and φ30 μm. As seen in the figure, for probes of φ50 μm and φ30 μm, resist films were partially damaged after contact tests (shown inside the red circles in the figure) so that area-control was no longer achieved.

**Figure 9:**
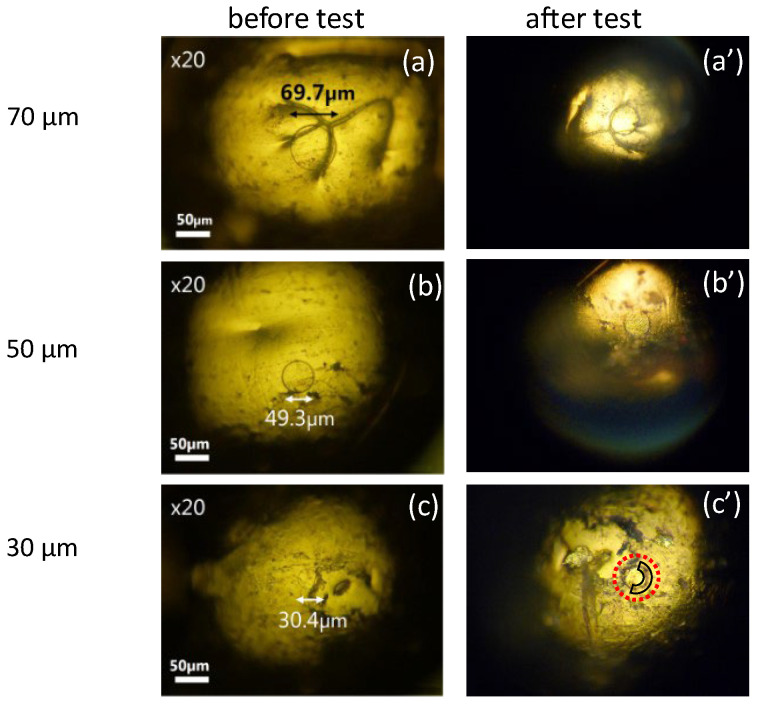
Photos of area-controlled (φ70, φ50, and φ30 μm) probes with laser lithography using process #2 before (**a**–**c**) and after (**a’**–**c’**) and robustness test. Inside the red circles, resist films are partially damaged.

The test results of probes fabricated with process #2 (pre-baking at 110 °C and no post-treatment) are shown in Figure 9. Here, photos before the robustness test are labeled (a), (b), and (c) for φ70 μm, φ50 μm, and φ30 μm, respectively. Those after the test are labeled with (a’), (b’), and (c’) for the corresponding sizes. For probes of φ30 μm, resist films were partially damaged after contacts, as shown inside the red circle, so area-control was no longer achieved. For probes of φ50 μm or larger, robust area-controlled probes were fabricated.

**Figure 10:**
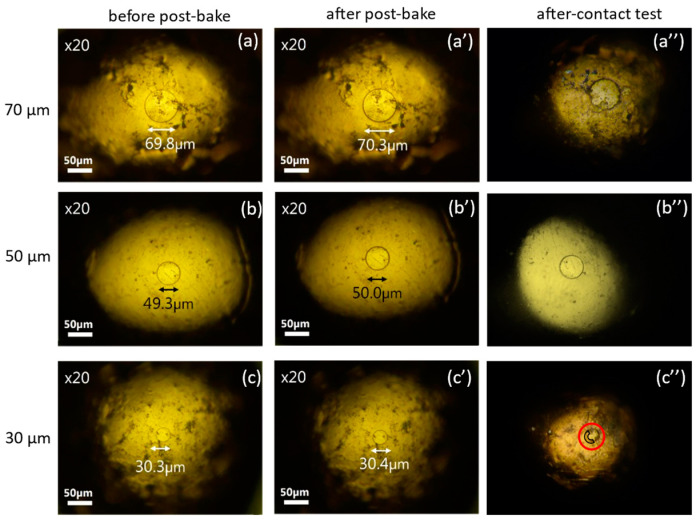
Photos of area-controlled (φ70, φ50, φ30 μm) probes with laser lithography using process #3. Probes pre-baked at 110 °C and used in lithography are labeled as (**a**–**c**). Those pre-baked at 110 °C and used in lithography and post-baked at 110 °C are labeled as (**a’**–**c’**). After-contact test photos (**a’’**–**c’’**) refer to photos taken after the probes underwent several electrical contact tests. Inside the red circles, resist films are partially damaged.

Figure 10 demonstrates examples of the robustness test results of probes fabricated with process #3 (pre-baking at 110 °C and post-baking at 110 °C). Here, the photos before post-bake (after lithography, labeled a, b, and c), after post-bake (a’, b’, and c’), and after robustness contact test (a’’, b’’, and c’’) are demonstrated for each size. Although the resist film was partially damaged for the φ30 μm probe, as seen inside the red circle in the figure (c’’), robust area-controlled probes were fabricated for probes of φ50 μm or larger.

**Figure 11:**
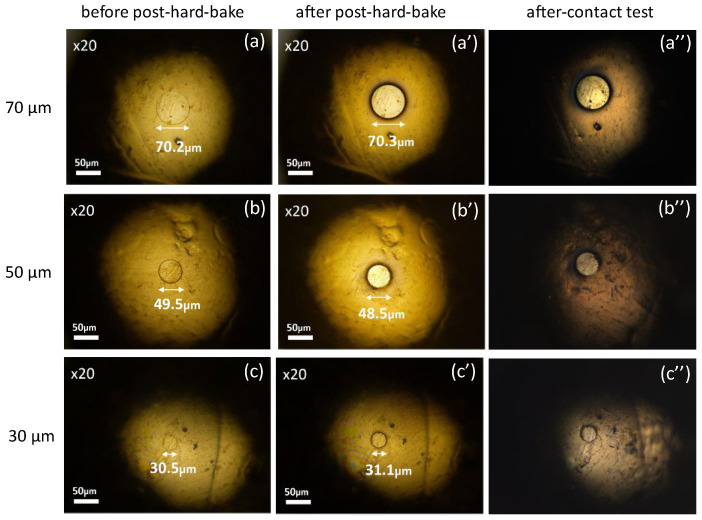
Photos of area-controlled (φ70, φ50, and φ30 μm) probes with laser lithography using process #4 before post-hard-bake (pre-baked at 110 °C and lithography, denoted as **a**–**c**), after post-hard-bake at 180 °C (**a’**–**c’**), and after-contact test (**a’’**–**c’’**).

So far, robust area-controlled probes of φ30 μm have not been achieved. Therefore, we tried the combination of pre-baking at 110 °C for 10 min and post-hard-baking at 180 °C for 20 min (process #4 in Table 1). The photos before post-bake, after post-hard-bake, and after-contact test are demonstrated in Figure 11. While post-hard-baking prevented the peeling or damaging of the resist film even for a φ30 μm probe (c’’), post-hard-baking caused the change in the color at the circular edge of the resist film for all sizes of the probes (a’, b’, and c’), indicating that the resist film got burned. Then, the surface of the resist became hard, and soft materials were easily damaged, which is not desirable for contact with fragile specimens. Therefore, we fabricated area-controlled probes without post-hard-baking but with post-baking (process #3 in Table 1).

In Table 2, the effect of different treatments before and after laser exposure on the quality of area-controlled probes for φ70 μm, φ50 μm, and φ30 μm probes are summarized.

**Figure 12:**
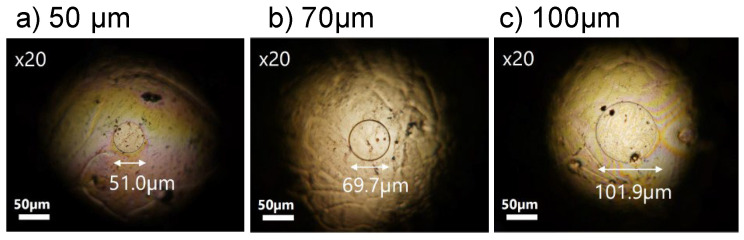
Examples of optical photos of area-controlled Pt-coated probe fabrication by laser lithography with process #3.

Although we systematically examined the effect of treatments on the robustness of resist films on Au probes, the results were similar for Cu- and Pt-coated probes. Figure 12 displays optical micrographs of the lithographed probes of Pt-coated ones.

### 4.2. Examples of Measurements with the Area-Controlled Probes

**Figure 13:**
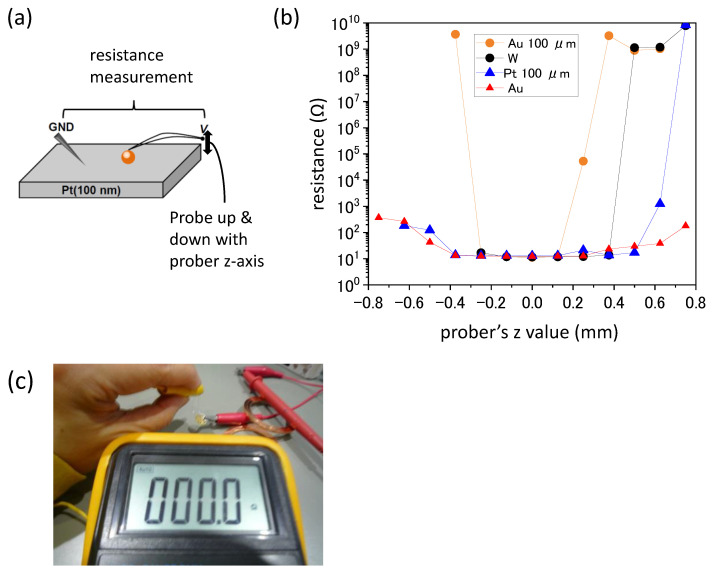
The setup of the resistance measurements on 100 nm Pt films with the area-controlled probes using a prober (**a**). The results of resistance measurements using φ100 μm Au probe, φ100 μm Pt probe, non-area controlled Au probe, and commercially available ordinary W needle probe (**b**). Resistance measurement with φ100 μm Au probe using a conventional multi-meter (**c**).

Figure 13a illustrates the setup of the resistance measurements on 100 nm Pt films with the area-controlled probes using a prober. In addition to the area-controlled probes of Au- and Pt-coated, one non-area-controlled Au probe and commercially available ordinary W needle probe were used for the measurements [52]. With the prober’s Z-axis micrometer, probes can be pushed toward the Pt film or lifted from the Pt film. The resistance between the probe and GND shown in Figure 13a was measured as a function of the prober Z-axis value, which is depicted in Figure 13b. From the minus value of z to zero, probes were approaching and pushing toward the Pt film, while probes were lifting from the Pt film from zero to a plus value. In the region with the lowest resistance values around z = 0, the probes were contacting with the Pt film. The lowest resistance in the figure is not zero, but this is considered either due to thin Pt film or circuit resistance (resistance with W needle shows the same lowest resistance). In fact, when resistance was measured with a φ100 μm Au probe using a conventional multi-meter, resistance showed zero [51], as depicted in Figure 13c. Since the contact area changes by changing the pushing force (in this case, the prober Z-axis change) with a non-area-controlled Au probe, low resistance is kept with a much wider Z-axis value than with φ100 μm Au probe. With a φ100 μm Pt probe, low resistance is kept with a slightly wider Z-axis value than with a φ100 μm Au probe, possibly because Pt has a larger elastic constant (larger force needed for elastic deformation).

**Figure 14:**
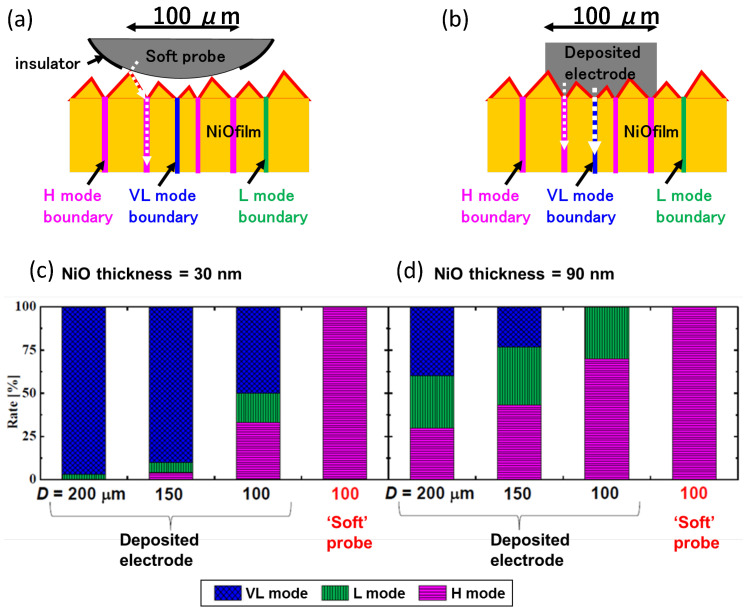
Evaluation of ReRAM function with Pt-coated φ100 μm probe (**a**). Evaluation of ReRAM function with deposited electrode of the same size (**b**). Comparison of measured switching behavior between deposited electrode and Pt-coated φ100 μm soft probe on 30 nm-thick (**c**) and 90 nm-thick (**d**) NiO films.

Figure 14 demonstrates the advantage of the developed soft probe over the electrode deposition method for studies of NiO Resistive Random Access Memory (ReRAM). ReRAM functions as a switch when a bias voltage is applied, with a phase change between the conductive phase and the non-conducting phase upon voltage application. Therefore, short-circuiting should be avoided. The H-mode grain boundaries of NiO columnar structure shown in Figure 14a,b works as ReRAM [53]. However, there are VL-mode boundaries and L-mode boundaries, which are conductive and do not function as ReRAM. The surface of each grain works as a H-mode. When a Pt-coated φ100 μm probe was used for the evaluation of switching behavior, the NiO film functioned 100% as ReRAM, as seen in the right side of Figure 14c,d in purple color both for 30 nm and 90 nm-thick NiO films. However, when a deposited electrode with a diameter of 100 μm (Figure 14b, the same contact area as the Pt-coated φ100 μm probe) was used, deposited electrode metal went into the grain and touched either VL or L-mode grain boundary, resulting in short-circuiting with 70% and 30% probability for 30 nm and 90 nm-thick NiO film as seen in Figure 14c,d in blue and green color. Very similar ReRAM functions were also reported on CoO films [54].

## 5. Newly Developed Area-Controlled Probe

Although the lithography method was successful in fabricating an area-controlled probe, it proved costly and impractical. Additionally, the concave contact area posed difficulties in establishing an electrical connection. Therefore, we have developed a more economical method to fabricate an area-controlled probe.

### 5.1. Fabrication

**Figure 15:**
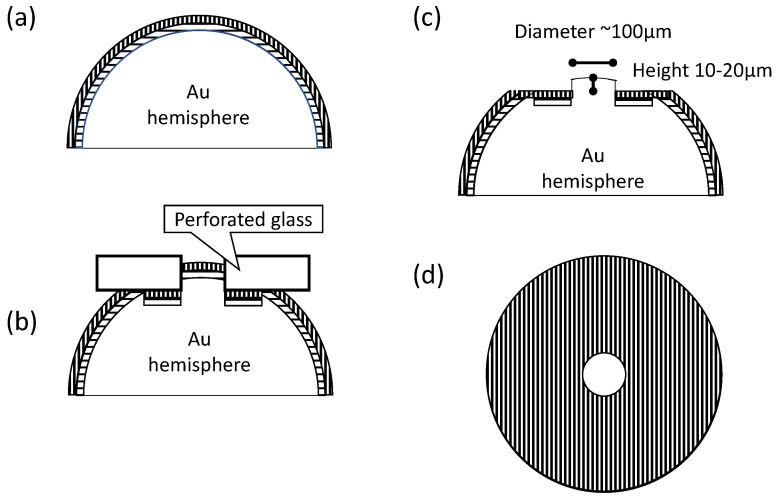
Process of area-controlled probe fabrication. (**a**) dip-coating of polyvinyl alcohol and resist (nail polish), (**b**) pushing a perforated glass on the coated probe, (**c**) after washing (**b**), (**d**) resist-coated area hatched.

Instead of using lithography, we employed a glass plate with a hole [55] as a mold. Figure 15a,b illustrate the process of fabricating the contact area. Initially, polyvinyl alcohol was dip-coated and dried, followed by a layer of nail polish, which served as an insulator [56,57,58]. A glass plate with a hole of φ100 μm was then pressed onto the coated Au hemisphere, creating a convex top area on the ball. Subsequently, the embossed ball underwent supersonic washing in water for 10 s. Since polyvinyl alcohol is water-soluble, it dissolved, separating the Au ball from the nail polish layer at the convex portion, thus exposing the Au ball, which functions as the electrical contact.

**Figure 16:**
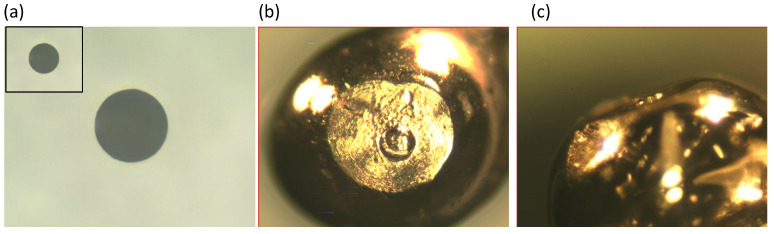
A photograph of a perforated glass with a φ100 μm hole (**a**) with the same magnification of (**b**) and (**c**) at the top-left square. The top-view (**b**) and side-view (**c**) photographs of a probe that was deformed by a perforated glass being pushed.

The one example photograph of a perforated glass with a φ100 μm hole is shown in Figure 16a. To show the good shape of the hole and the smoothness of the hole edge, this photo is magnified larger than the photos of the fabricated probe using the glass (Figure 16b,c). In the upper-left of Figure 16a, the magnification is adjusted to the photos of the fabricated probe. Figure 16b,c display the top-view and side-view photographs of a probe that was deformed by a perforated glass being pushed. It is clear that the convex is formed with a height of 10–20 μm, as designed in Figure 15c.

### 5.2. Resistance Measurements

The electrical contact was measured using a conventional electric multi-meter by pressing the fabricated probe against a gold plate and measuring the resistance between the probe’s support and the gold plate in a similar way to Figure 13c. The resistance was found to be either zero or infinite, depending on the angle of contact, indicating that the electric resistance is zero when the convex part is in contact, while the resistance is infinite (shown as OL = overload) for the contact with the surrounding area. These results clearly suggest that the contact area on the probe is restricted.

**Figure 17:**
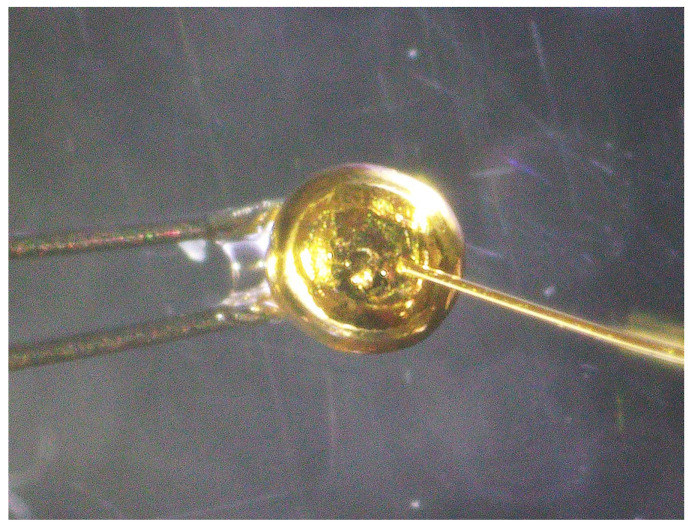
An example of views under the low magnification microscope where a φ50 μm gold wire touches the flat area of the area-controlled probe, which is covered by the resist film.

To confirm that the electric contact was limited to the convex part of the probe, resistance measurements were conducted between the support wire and a thin gold wire touching either the convex part or the flat area around it under low magnification microscopy. Figure 17 demonstrates an example of views under the microscope, where a φ50 μm gold wire touches the flat area of the probe covered with the resist film. Under this condition, resistance was infinite (OL). Resistance was zero when the gold wire touched the convex part. We measured resistance on many different positions of flat areas and of convex parts and obtained the same results as mentioned above. These results confirm that only the convex part is conductive and the surrounding flat area is insulating.

The convex type of area-controlled probes is much easier to handle and find conductive areas. Although there are no further examples of electric measurements for practical materials, demonstrating the establishment of area-control should be enough for the use.

### 5.3. Analysis of Conducting Area

**Figure 18:**
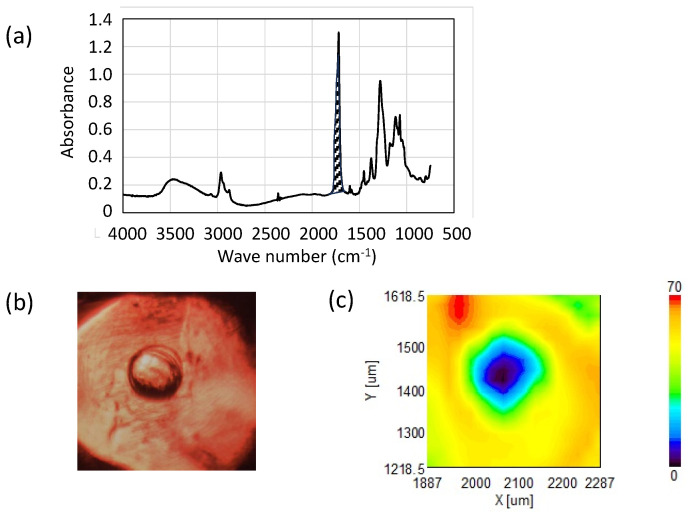
(**a**) IR spectra of resist-coated area, (**b**) optical image of the area-controlled probe, (**c**) intensity mapping of IR peak area hatched in (**a**), corresponding to the resist material.

To visualize the area not covered by insulating film (nail polish), we employed microscopic FTIR analysis (Jasco FT/IR-6100). Figure 18a presents the FTIR spectra from a flat area of a fabricated Au probe measured with a 25 μm slit. An optical micrograph of the probe taken with infrared light is shown in Figure 18b. The area intensity hatched around 1740 cm^−1^ in Figure 18a, corresponding to the C=O stretching mode of the ester group [59,60], was mapped to illustrate the nail polish’s presence. Figure 18c presents this IR area intensity mapping.

**Figure 19:**
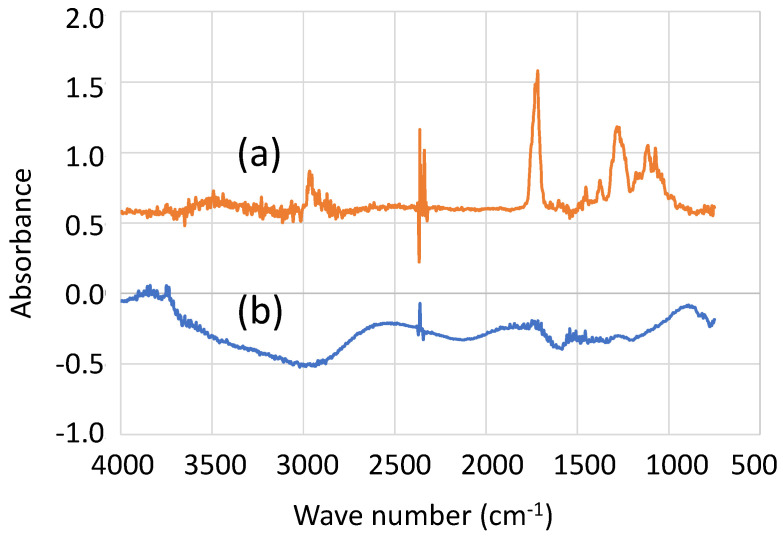
IR spectrum with 10 μm slit (**a**) on the flat area and (**b**) on the convex.

It is evident that the nail polish was removed at the convex center while it remained on other parts of the probe, providing insulation. To further verify the removal of nail polish at the convex center, IR spectra with a 10 μm slit were measured. Figure 19 compares IR spectra with 10 μm slits (a) on a nail polish-covered area and (b) on the convex. Compared with Figure 18a, the 10 μm slit spectra were noisier, but the 1740 cm^−1^ peak was distinctly observed on the flat, nail polish-covered area (a), while it was obscured by noise and undetectable on the convex (b).

### 5.4. Robustness Test

**Figure 20:**
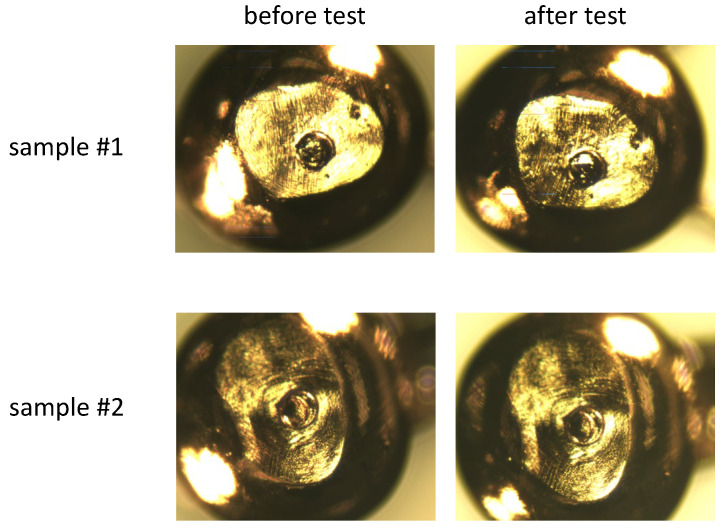
Photographs of probes with a φ100 μm concave before and after several times of electrical contact.

The robustness of the resist coatings against electrical contact is tested by comparing optical micrograph images before and after several contacts. For probes fabricated with the new method, good robustness is expected since the contacting area has a convex shape, as seen in Figure 16c, so the resist film around it is not under force. The results for the two samples are shown in Figure 20. There is no sign of partial peeling or damage. An electrical contact test using a gold plate also demonstrated that depending on the angle of touching, the concave part is conductive, but the surrounding area of the concave is insulating.

All the above results demonstrate successful contact area control using this new fabrication method with a glass plate and hole. We also applied this method to fabricate a convex with a 50 μm diameter. However, the quality of the hole in the glass affected the convex shape, resulting in a poorer quality compared to the 100 μm diameter convex. In principle, this method can be used to fabricate smaller convex shapes. We tried applying the fabrication process in Figure 15 using a perforated glass with φ70 μm and φ50 μm holes [61]. However, the quality of the resulting area-controlled probe has not been good enough. The biggest reason is that the quality of the shape and the edge sharpness of the holes are poorer for φ70 μm and φ50 μm compared to φ100 μm holes, resulting in the non-circular shape of convex and incomplete removal of resist films.

We used perforated glass as a mold since a transparent material for a mold is necessary to monitor the position of the probe and the hole by a low-magnification optical microscope during the fabrication of a convex (process (b) in Figure 15). However, if more sophisticated methods to monitor the position of the probe and the hole (to push the hole to the top of the hemisphere of the probe) are applied, other materials, such as metal, can be used. We confirmed a good-shaped convex formation using an aperture for an electron microscope (even as small as φ30 μm), though the control of the position of convex forming was difficult without optical monitoring.

## 6. Summary

This paper presents the fabrication and characterization of contact-area-controlled probes. Following several approaches, we successfully established a method to fabricate low-cost, area-controlled probes for electrical contact, which avoids damage to fragile specimens. The control over the contact area was verified using infrared (IR) spectra and Fourier transform infrared (FTIR) imaging. These probes are particularly useful for electrical property measurements where the contact area is critical, such as in capacitance measurements and measurements conducted under constant electric current.

## Figures and Tables

**Table 1:** Various processes with different pre-treatments and post-treatments.

	Pre-Treatment (after Dip Coating)	Post-Treatment (after Lithography)
Process #1	vacuum dry	no
Process #2	pre-bake	no
Process #3	pre-bake	post-bake
Process #4	pre-bake	post-hard-bake

**Table 2:** Difference in the robustness of electrical contacts with different treatments.

	Vacuum Dry	Pre-Bake	Pre-Bake + Post-Bake	Pre-Bake + Post-Hard-Bake
φ70 μm	good	good	good	black
φ50 μm	bad	good	good	black
φ30 μm	bad	better than bad	good or better than bad	black

## Data Availability

Data are contained within the article.
